# The Inhibition of CDK8/19 Mediator Kinases Prevents the Development of Resistance to EGFR-Targeting Drugs

**DOI:** 10.3390/cells10010144

**Published:** 2021-01-12

**Authors:** Amanda C. Sharko, Chang-Uk Lim, Martina S. J. McDermott, Chuck Hennes, Kingsavanh P. Philavong, Tiffanie Aiken, Victor V. Tatarskiy, Igor B. Roninson, Eugenia V. Broude

**Affiliations:** 1 Department of Drug Discovery and Biomedical Sciences, University of South Carolina, Columbia, SC 29208, USA; sharko@cop.sc.edu (A.C.S.); limc@cop.sc.edu (C.-U.L.); martinamcdermott3@gmail.com (M.S.J.M.); ChuckHennes55@gmail.com (C.H.); philavong.kp@gmail.com (K.P.P.); taiken0518@gmail.com (T.A.); roninsoni@cop.sc.edu (I.B.R.); 2 Institute of Gene Biology, Russian Academy of Sciences, 119334 Moscow, Russia; tatarskii@gmail.com

**Keywords:** CDK8, CDK19, EGFR, gefitinib, erlotinib, cetuximab, drug resistance

## Abstract

Drug resistance is the main obstacle to achieving cures with both conventional and targeted anticancer drugs. The emergence of acquired drug resistance is initially mediated by non-genetic transcriptional changes, which occur at a much higher frequency than mutations and may involve population-scale transcriptomic adaptation. CDK8/19 kinases, through association with transcriptional Mediator complex, regulate transcriptional reprogramming by co-operating with different signal-responsive transcription factors. Here we tested if CDK8/19 inhibition could prevent adaptation to drugs acting on epidermal growth factor receptor (EGFR/ERBB1/HER1). The development of resistance was analyzed following long-term exposure of BT474 and SKBR3 breast cancer cells to EGFR-targeting small molecules (gefitinib, erlotinib) and of SW48 colon cancer cells to an anti-EGFR monoclonal antibody cetuximab. In all cases, treatment of small cell populations (~10^5^ cells) with a single dose of the drug initially led to growth inhibition that was followed by the resumption of proliferation and development of drug resistance in the adapted populations. However, this adaptation was always prevented by the addition of selective CDK8/19 inhibitors, even though such inhibitors alone had only moderate or no effect on cell growth. These results indicate that combining EGFR-targeting drugs with CDK8/19 inhibitors may delay or prevent the development of tumor resistance to therapy.

## 1. Introduction

Cancer drug resistance, a key obstacle to achieving cures, was long thought to result from the selection of rare preexisting tumor cells that carry genetic changes conferring resistance. In recent decades, however, it became clear that resistance to both conventional and targeted drugs involves metastable transcriptional changes that allow tumor cells to adapt and survive drug exposure [1,2,3,4,5,6,7,8]. While conclusions from different studies using the same drugs have varied in regard to the relative contributions of pre-existing genetic and stable epigenetic variants towards drug resistance [9,10,11,12,13], a consensus is emerging that transcriptomic plasticity of tumor cells and drug-induced adaptation is key to the initial emergence of low-level resistance. This non-genetic resistance of tumor cell populations provides the background for subsequent selection of stable genetic variants that yield higher levels of resistance [5,13].

Most importantly from the therapeutic perspective, the induction of non-genetic resistance was found to be preventable pharmacologically, which has been first demonstrated by the ability of PKC/NFκB pathway inhibitors to prevent non-genetic induction of the multidrug transporter ABCB1 (MDR1/Pgp) [1,2]. In more recent examples, resistance to different classes of receptor tyrosine kinase inhibitors was reported to be prevented by THZ1, an inhibitor of transcriptional kinases CDK7, CDK12 and CDK13 [14], whereas a BRD9 inhibitor was shown to prevent resistance to EGFR-targeting agents [15] and a compound that prevented tamoxifen resistance was found to act by inhibiting NFκB [16]. However, agents that affect the general transcriptional machinery or inhibit basal NFκB activity are usually cytotoxic, which complicates both the study interpretation (independent cytotoxicity versus prevention of resistance) and the future clinical development.

We are exploring the potential utility of a new class of non-cytotoxic inhibitors of CDK8/19 Mediator kinases that regulate transcriptional reprogramming as novel resistance-preventing agents. CDK8 (ubiquitously expressed) and CDK19 (expressed in a tissue-specific manner) [17] are alternative components of the regulatory CDK8 module associated with the transcriptional Mediator complex; this module also includes cyclin C (CCNC, the binding partner of CDK8 and CDK19) and proteins MED12 and MED13 [18]. Unlike better-known CDKs (such as CDK2 or CDK4/6), CDK8/19 regulate transcription but not cell cycle progression [18,19]. Unlike other transcriptional CDKs, such as CDK7 or CDK9, they do not affect the overall transcription machinery. While CDK8 knockout is detrimental to embryonic development, a process that relies on transcriptional reprogramming [20,21,22], the knockout of CDK8 in adult mice has no apparent phenotypic consequences [23] and the knockdown or knockout of CDK8 or CDK19 [24] or pharmaceutical CDK8/19 kinase inhibitors [25,26,27] have little or no effect on the growth of most cell lines, in contrast to the effects of CDK7, CDK9 or cell cycle regulating CDKs [24]. Instead, CDK8/19 act as cofactors of several transcription factors, including β-catenin/TCF/LEF [28], SMADs [29,30], HIF1A [31], STATs [32], ER [33] and NFκB [34]; CDK8/19 inhibition decreases the induction of transcription of subsets of genes induced by the above transcription factors. CDK8/19 act both by direct phosphorylation of some transcription factors (e.g., SMADs, STATs) and also downstream of transcription factors by affecting (possibly indirectly) the phosphorylation of the C-terminal domain (CTD) of RNA polymerase II (Pol II), which allows the paused Pol II to detach from the promoter and complete transcription. The CTD phosphorylation-based mechanism was shown to be responsible for downstream potentiation of the serum response network [35], HIF1A [31], estrogen receptor (ER) [33] and NFκB [34] by CDK8/19. Importantly, CDK8/19 inhibition suppresses CTD phosphorylation not globally but only in the context of newly activated genes and CDK8/19 inhibitors suppress de novo induction of gene expression but not basal transcriptional activity [34]. This unique transcription regulatory pattern defines CDK8/19 as regulators of transcriptional reprogramming [34].

The first evidence for the role of CDK8/19 in drug resistance came from a chemical genomics study where CDK8/19 were identified as the targets of senexin A and related small molecules that suppressed chemotherapy- or radiation-induced transcription of multiple paracrine factors that inhibit apoptosis and confer drug resistance [25]. These effects of CDK8/19 inhibitors were shown to be due to a large extent to the attenuation of NFκB-induced transcription [34]. In another study, we found that CDK8/19 inhibitors senexin A and senexin B prevent the development of estrogen independence in three different estrogen receptor-positive breast cancer cell lines during long-term estrogen deprivation [33].

In the present study, we tested the ability of highly selective chemically distinct CDK8/19 inhibitors senexin B [34,36,37] and 15w [36,37] to prevent resistance to drugs targeting the epidermal growth factor receptor (EGFR/ERBB1/HER1). We have analyzed the development of resistance to small-molecule EGFR inhibitors gefitinib and erlotinib, which are used in non-small-cell lung cancers with mutated EGFR and that have been tested in other EGFR-driven tumors including breast cancers [38,39,40,41] and cetuximab, an anti-EGFR monoclonal antibody used for the treatment of colorectal cancer (with wild-type KRAS), non-small cell lung cancer and head and neck cancer [42]. Cancers treated with all the EGFR-targeted drugs eventually become resistant to them [43] indicating that prevention of such resistance is an important medical need. We have analyzed the effects of CDK8/19 inhibitors on the development of adaptive resistance in cell lines derived from breast cancer (gefitinib, erlotinib) and colon cancer (cetuximab). In all cases, the CDK8/19 inhibitors, while showing moderate or no growth-inhibitory activity when used alone, strongly suppressed the development of resistance to the EGFR-targeting agents. These results suggest that CDK8/19 inhibition may delay or prevent the development of resistance to EGFR-targeting drugs.

## 2. Materials and Methods

### 2.1. Cell Lines and Reagents

BT474, SKBR3 and SW48 cells were obtained from the American Type Culture Collection (Manassas, VA, USA). BT474 (ATCC: HTB-20) and SKBR3 (ATCC: HTB-30) cells were maintained, respectively, in RPMI-1640 and DMEM (ThermoFisher Scientific, Waltham, MA, USA) with 10% FBS, 1% penicillin-streptomycin and 2 mM l-glutamine. SW48 (ATCC: CCL-231) were cultured in RPMI-1640 and 10% FBS. Cells were routinely confirmed to be free of Mycoplasma (MycoAlert PLUS mycoplasma detection kit, Lonza, Walkersville, MD, USA) and were authenticated by STR profiling by the University of Arizona Genetic Core or by Source Bioscience in 2016. Gefitinib and erlotinib were purchased from LC Laboratories (Woburn, MA, USA), cetuximab (Erbitux) was from ImClone/Eli Lilly (Indianapolis, IN, USA), and senexin B and 15w from Senex Biotechnology (Columbia, SC, USA).

### 2.2. Gefitinib and Erlotinib Selection and Sensitivity Testing

To determine concentrations to select for acquired drug resistance, BT474 and SKBR3 breast cancer cells were seeded in 96-well plates (2500 and 1500 cells/well, respectively) and treated with gefitinib (0–5 µM) and (in the case of BT474) with erlotinib (0–10 µM) for 10 days. Visual assessment and image densitometry were used to determine the approximate minimum concentration necessary to produce maximum growth inhibition. Drug resistant cells were selected by plating 150,000 cells in T25 flasks and treating continuously with either erlotinib (BT474: 7.5 µM) or gefitinib (BT474: 1.25 µM; SKBR3: 2.0 µM), passaging as necessary. Cells were concurrently treated with erlotinib or gefitinib in combination with senexin B (1 µM) or 15w (250 nM). Cell cultures were photographed in situ (Zeiss Axiovert 200; Carl Zeiss Microscopy, White Plains, NY, USA) before treatment, at 3 days, and then once weekly for 8–10 weeks after treatment initiation. Densitometric measurements were made using ImageJ software. Statistics were calculated by 2-way ANOVA with post-hoc multiple comparisons in Prism (GraphPad Software, San Diego, CA, USA). Representative flasks were also fixed, stained with crystal violet, and photographed at 2, 4, and 8–10 weeks after initiation of treatment.

For assessing drug resistance, BT474 and SKBR3 cells were seeded in 96-well plates (2500 and 1500 cells/well, respectively) and incubated overnight. Cells were then treated with DMSO (control), senexin B (0–3 µM), erlotinib (0–15 µM), or gefitinib (0–3 µM) individually or in fixed ratio combinations (1:1 for gefitinib and senexin B and 5:1 for erlotinib and senexin B). Cell growth was measured at 7 days after drug addition by MTT assay and expressed as a percentage of untreated cell signal. Synergy was assessed by calculating Combination Indices (CI values) using CompuSyn Software [44] and IC50 values were determined using the AAT Bioquest IC50 calculator (https://www.aatbio.com/tools/ic50-calculator/).

### 2.3. Cetuximab Selection and Sensitivity Testing

SW48 cells were seeded in 6-well plates (40,000 cells/well) and treated with cetuximab (0–1 µM) and senexin B (0.313–5 μM) for 7 days. The number of live cells was measured by flow cytometry using a LSR II system (Becton Dickinson, Franklin Lakes, NJ, USA) with a 96-well loader, after staining with 10 μg/mL propidium iodide. For long-term selection, parental SW48 cells were plated in T75 flasks (500,000 cells/flask) and then treated with vehicle (DMSO), or with cetuximab (100 nM) and senexin B (1 µM) alone and in combination. Cells (in duplicates) were collected, live cell numbers were measured by flow cytometry and cells were replated at the original density every 6–10 days for a total of 81 days. Changes in cell number over time were calculated relative to vehicle-treated cells using a multiplying factor (the ratio of the number of cells collected to the number plated at each passage).

## 3. Results

### 3.1. Effects of CDK8/19 Inhibition on Gefitinib and Erlotinib Resistance

**Figure 1:**
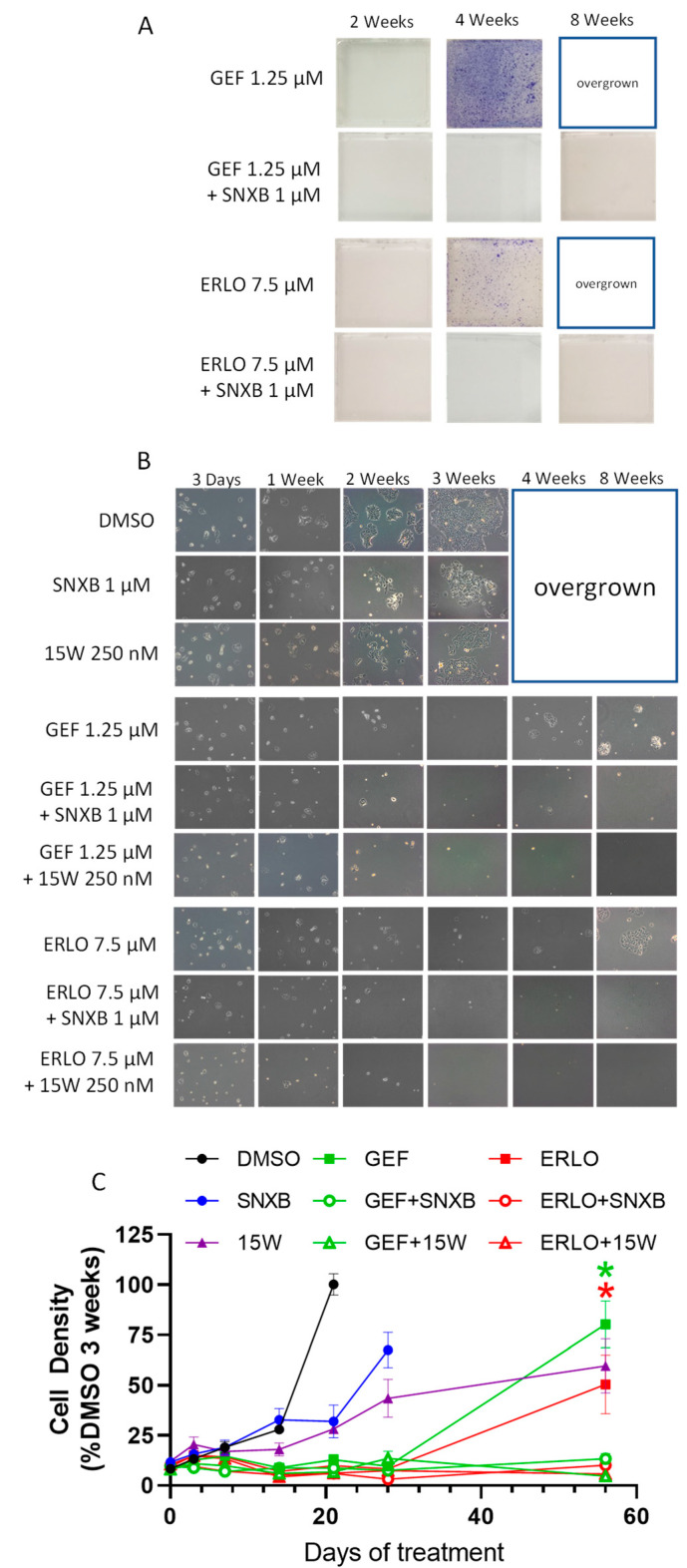
CDK8/19 inhibitors senexin B (SNXB) and 15w prevent resistance to EGFR inhibitors gefitinib (GEF) and erlotinib (ERLO) in BT474 breast cancer cells. (**A**). Representative photographs of crystal violet-stained flasks at 2, 4, and 8 weeks of treatment. (**B**). Representative phase-contrast microphotographs at 3 days, and at 1, 2, 3, 4 and 8 weeks of treatment. (**C**). Densitometric measurements of microphotographs expressed as percentage of cell density in DMSO controls at 3 weeks. Data shown as mean (*n* = 4 images/flask) ± SEM. *p* < 0.0001 for GEF vs. GEF+SNXB/15w (*) and ERLO vs. ERLO+SNXB/15w (*) at 8 weeks.

We used breast cancer cell lines to analyze the effects of CDK8/19 inhibitor senexin B on the development of resistance to small-molecule EGFR inhibitors gefitinib and erlotinib. Single-step selections of limited duration (2–3 months) were carried out starting from 1.5 × 10^5^ cells. This starting cell number and duration of exposure are too low to select drug-resistant mutants (see **Discussion**). Cells were plated in the presence of gefitinib or erlotinib at concentrations that produced near-maximum inhibition of cell growth after 10 days (see **Materials and Methods**). The results of representative single-step selections (from a total of three independent gefitinib and three erlotinib selections) conducted in BT474 breast cancer cells (ER- and HER2-positive) are shown in Figure 1. Figure 1A shows crystal violet staining of representative flasks at three time points of selection. Figure 1B shows phase contrast microscopic images of cells at multiple time points and Figure 1C presents densitometric measurements of microphotographs (four images per flask). After two weeks of continuous treatment, cells were nearly undetectable by crystal violet staining (Figure 1A) and very few cells were detectable microscopically (Figure 1B,C). After an additional two weeks of treatment (four weeks total), cells resumed proliferation and were actively growing at the 8-week time point, indicating that the cells had adapted to EGFR inhibitors.

To test the effect of CDK8/19 inhibition on the outcome of selection, we have used the compound senexin B (4-((2-(6-(4-methylpiperazine-1-carbonyl)naphthalen-2-yl)ethyl)amino)quinazoline-6-carbonitrile), which is highly selective for CDK8/19 based on the lack of off-target inhibition in extensive kinome profiling [45,46] and lack of phenotypic effects in CDK8/19 knockout cells [38,47]. In contrast, when selection was carried out in the presence of 1 μM senexin B (concentration sufficient for near-maximum CDK8/19 kinase inhibition in cell-based assays [33,46]), cells did not grow out even after 8 weeks and were undetectable by crystal violet staining (Figure 1A) or showed minimal numbers by phase contrast microscopy (Figure 1B,C). To confirm the effects of CDK8/19 inhibition on the development of EGFR inhibitor resistance, we employed a chemically unrelated CDK8/19 inhibitor, 15w (3-amino-4-(4-(4-(2-(dimethylamino)-2-oxoethyl)phenyl)-1,4-diazepan-1-yl)thieno [2,3-b]pyridine-2-carboxamide), which is also highly selective for CDK8/19 based on kinome profiling [36] and phenotypic analysis [37,46]. As with senexin B, the addition of 15w (used at 250 nM, due to its higher potency [38]) prevented the emergence of both gefitinib and erlotinib resistance, even after 8 weeks of treatment (Figure 1B,C), confirming that the resistance-preventing effect of senexin B was mediated by CDK8/19 inhibition.

**Figure 2:**
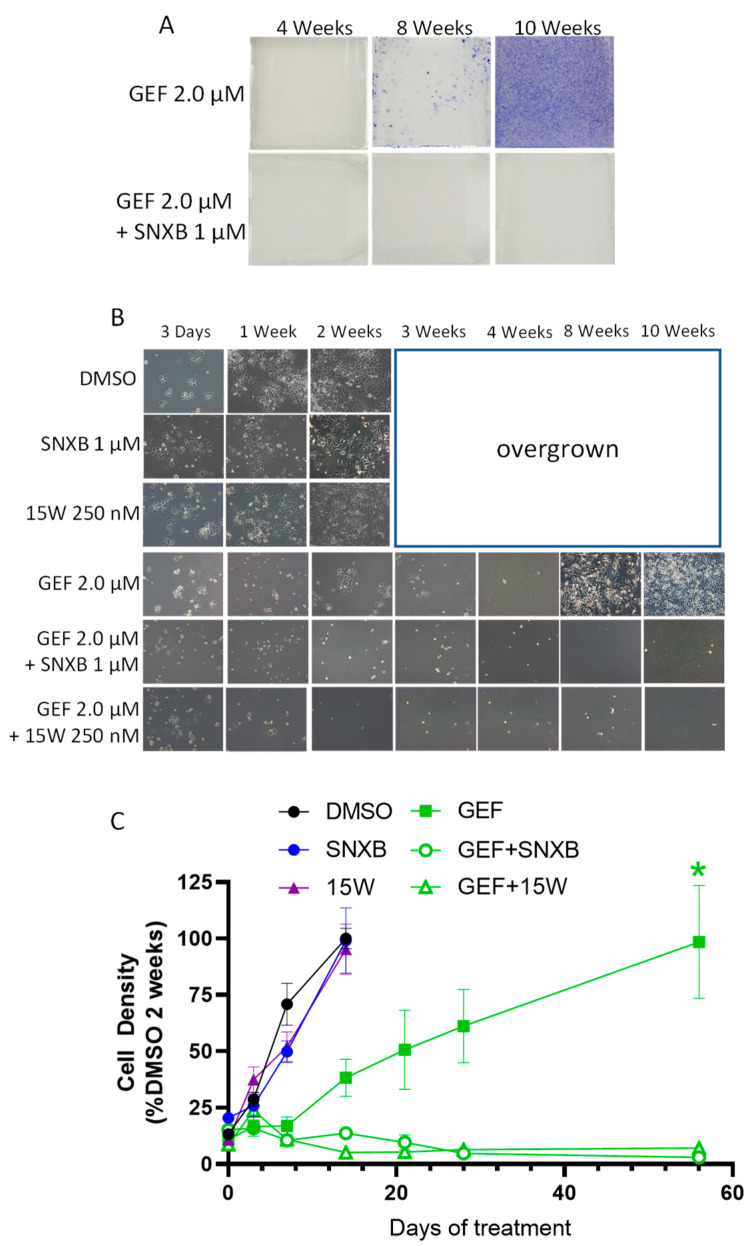
CDK8/19 inhibitors senexin B (SNXB) and 15w prevent resistance to EGFR inhibitor gefitinib (GEF) in SKBR3 breast cancer cells. (**A**). Representative photographs showing cell density (crystal violet staining) in flasks at 4, 8 and 10 weeks of treatment. (**B**). Representative phase-contrast microphotographs at 3 days, and at 1, 2, 3, 4, 8 and 10 weeks of treatment. (**C**). Densitometric measurements of photomicrographs expressed as percentage of cell density in DMSO controls at 2 weeks. Data shown as mean (*n* = 4 images/flask) ± SEM. *p* < 0.0001 for GEF vs. GEF+SNXB/15w (*) at 8 weeks.

To confirm the observed effects in another cell line, we have used SKBR3 breast cancer cells (ER-negative, HER2-positive) for gefitinib selection, using the same study design as with BT474 cells. Figure 2 shows the results of a representative gefitinib selection (out of 4 independent selections). Gefitinib resistance took longer to develop in SKBR3 cells than in BT474, but by 10 weeks cells appeared fully adapted to the drug (Figure 2A–C). As with BT474 cells, the development of resistance in SKBR3 cells was fully prevented by the addition of different CDK8/19 inhibitors, senexin B and 15w (Figure 2A–C).

We have asked if the prevention of gefitinib and erlotinib resistance by CDK8/19 inhibitors could be due either to synergy between EGFR-targeting drugs and CDK8/19 inhibitors or to the reversal of acquired resistance to gefitinib or erlotinib. Synergy analysis was carried out by the Chou-Talalay method [44], which compares the effects of different concentrations of drugs (gefitinib or erlotinib and senexin B) used individually or at fixed-ratio combinations. In this method, the drug interactions are characterized by the Combination Index (CI), where a synergistic interaction is defined by CI < 1. To determine if CDK8/19 inhibitor reversed the resistance acquired under our conditions, the same analysis was carried out on the gefitinib- or erlotinib-adapted cell populations, and the degrees of resistance to individual drugs and their combinations were determined by comparing IC50 values between the unselected and drug-adapted populations.

**Figure 3:**
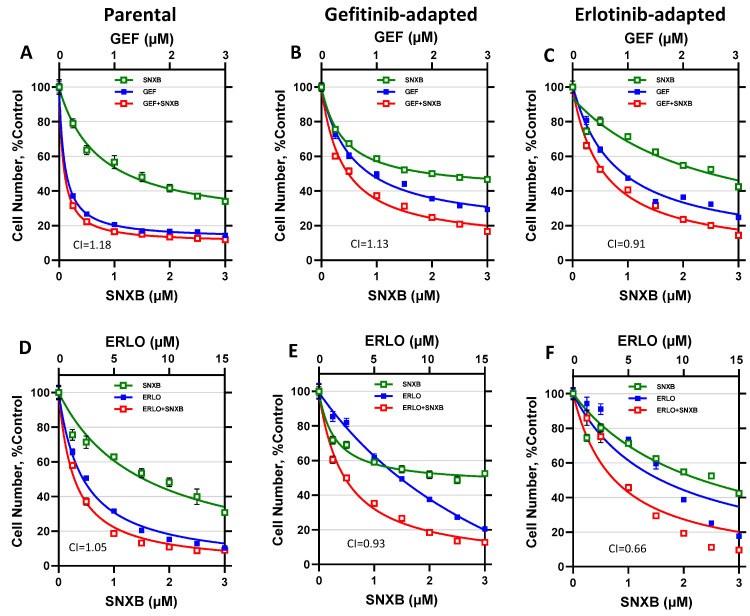
Effects of gefitinib and erlotinib alone and in combination with Senexin B in parental and gefitinib- or erlotinib-adapted BT474 cells. (**A**–**C**). Effects of 7-day treatment with gefitinib (GEF) and senexin B (SNXB), alone or in 1:1 combination, in parental (**A**), gefitinib-adapted (**B**), and erlotinib-adapted (**C**) BT474 cells. (**D**–**F**). Effects of 7-day treatment with erlotinib (ERLO) and senexin B, alone or in 5:1 combination, in parental (**D**), gefitinib-adapted (**E**), and erlotinib-adapted (**F**) BT474 cells. Data shown as mean ± SEM of six replicate measurements. Combination index (CI) values at IC50 concentrations are shown in lower left corner of each graph.

The analysis of gefitinib/senexin B interactions in BT474 cells is shown in Figure 3A–C and Table 1. Figure 3A shows the results of a 7-day growth inhibition assay of BT474 cells treated with gefitinib, senexin B, or their 1:1 combination. IC50 values measured in these assays are shown in Table 1 and CI values (determined at IC50 levels) are indicated in the graphs. Figure 3B,C and Table 1 show the results of the same analysis carried out with cells that were adapted to gefitinib (Figure 3B) or erlotinib (Figure 3C). Both gefitinib- and erlotinib-adapted BT474 cells showed increased gefitinib resistance (7.0-fold and 5.9-fold increase in IC50 relative to unselected cells, respectively). The addition of senexin B did not reverse resistance to EGFR inhibitors, as the resistant cells showed the same increase in IC50 values for gefitinib + senexin B combination relative to parental cells (5.9-fold for gefitinib-adapted and 7.0-fold for erlotinib-adapted cells). No significant synergy between gefitinib and senexin B was observed in parental cells or in cells selected with gefitinib or erlotinib (CI = 1.18, 1.13 and 0.91, respectively). These results indicate that the resistance-preventing effect of the CDK8/19 inhibitor was due neither to overcoming resistance that has already emerged nor to a synergistic interaction between the compounds.

The same analysis was carried out for erlotinib/senexin B interactions in BT474 cells. Figure 3D–F and Table 1 show the response of parental and drug-adapted BT474 cells to erlotinib, senexin B or their 5:1 combination. Both erlotinib- and gefitinib-adapted BT474 cells were resistant to erlotinib alone (3.4- and 2.9-fold relative to parental cells, respectively) and to erlotinib+senexin B combination (2.6- and 1.4-fold, respectively). CI values showed no synergy between erlotinib and senexin B for the parental and gefitinib-adapted cells (CI = 1.05 and 0.93, respectively) but this drug combination appeared synergistic (CI = 0.66) in erlotinib-adapted cells. Together with the finding that erlotinib-adapted cells were less resistant to erlotinib + senexin B combination than to erlotinib alone, this result suggests that reversal of erlotinib resistance could have contributed to the prevention of erlotinib resistance by CDK8/19 inhibitors.

**Figure 4:**
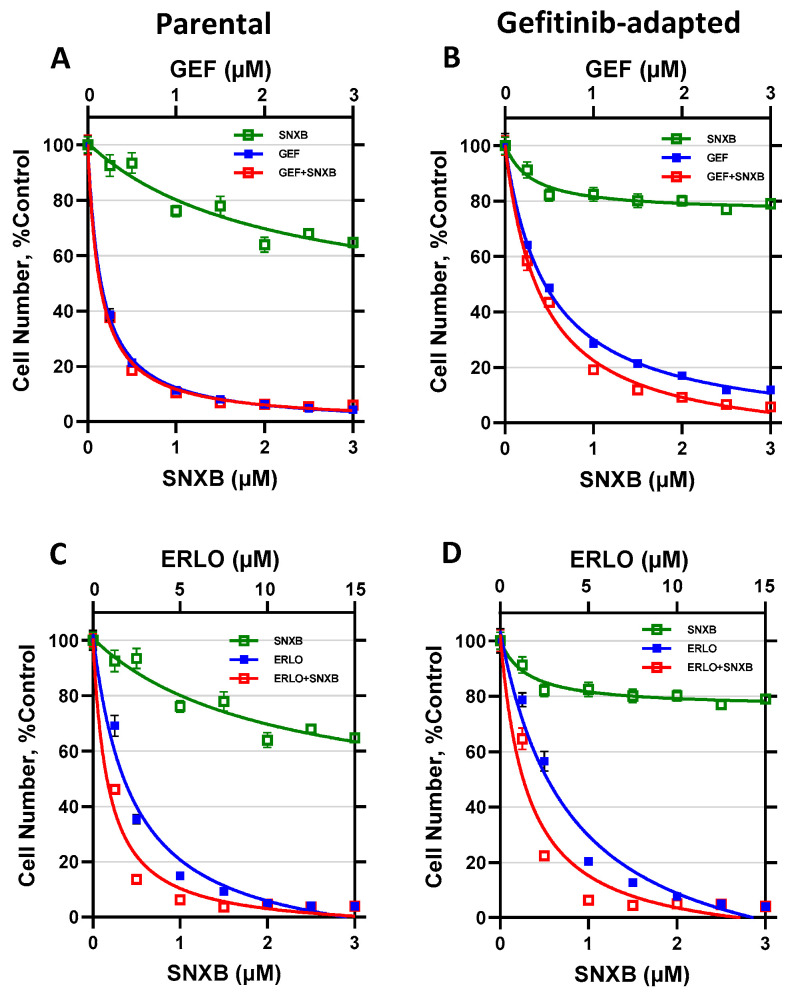
Effects of gefitinib and erlotinib alone and in combination with senexin B in parental and gefitinib-adapted SKBR3 cells. (**A**,**B**). Effects of 7-day treatment with gefitinib and senexin B, alone or in 1:1 combination, in parental (**A**) and gefitinib-adapted (**B**) SKBR3 cells. (**C**,**D**). Effects of 7-day treatment with erlotinib and senexin B, alone or in 5:1 combination, in parental (**C**) and gefitinib-adapted (**D**) SKBR3 cells. Data shown as mean ± SEM of six replicate measurements.

The same analysis for SKBR3 cells is shown in Figure 4 and Table 1. The effects of gefitinib and senexin B (Figure 4A,B) and erlotinib and senexin B (Figure 4C,D) are shown for the parental SKBR3 cells (Figure 4A,C) and their gefitinib-adapted population (Figure 4B,D). While senexin B showed significant growth inhibition in ER-positive BT474 cells (Figure 3), likely due to its activity as an inhibitor of estrogen signaling [33], the growth inhibitory effects of senexin B were very weak in ER-negative SKBR3 cells and never approached 50 percent. Hence, CI values at IC_50_ for the combinations of EGFR and CDK8/19 inhibitors could not be determined in SKBR3 cells. Gefitinib-adapted SKBR3 cells showed a moderate increase in their IC_50_ values for gefitinib (2.5-fold) and erlotinib (1.4-fold), whereas the increase in resistance to the combinations of these drugs with senexin B was 1.6-fold and 1.3-fold, respectively.

### 3.2. Effects of CDK8/19 Inhibition on Cetuximab Resistance

**Figure 5:**
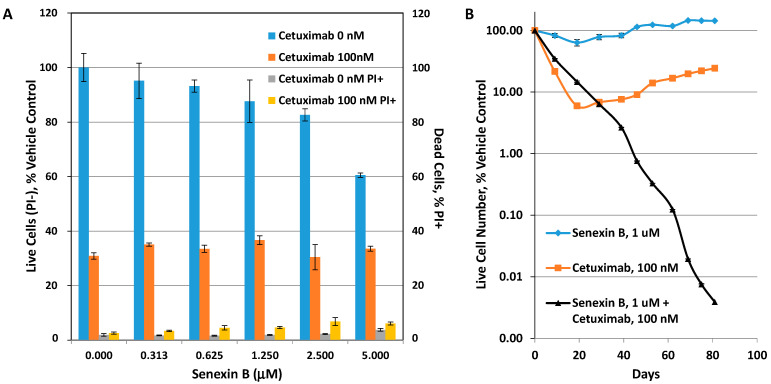
Effects of senexin B on the development of cetuximab resistance in SW48 colon carcinoma cells. (**A**) Effects of 7-day treatment with the indicated concentrations of Senexin B combined with vehicle or 100 nM cetuximab on the number of live (PI-) cells relative to vehicle control and the fraction of dead (PI+) cells. (**B**) Effects of prolonged treatment with senexin B (1 μM), cetuximab (100 nM) or senexin B plus cetuximab combination. Cell numbers for each time point are shown relative to untreated cells (mean ± SEM of duplicate measurements; error bars at most points are smaller than symbol size).

Cetuximab selection was carried out in SW48 colon carcinoma cells (wild-type KRAS), using 100 nM cetuximab. As shown in Figure 5A, this concentration decreases the number of live (propidium iodide (PI)-negative) SW48 cells to 30.9% of untreated cells after 7-day treatment, whereas the dead (PI-positive) cell fraction was below 3%, indicating that the effect of cetuximab is primarily cytostatic. The addition of senexin B at concentrations below 2.5 μM had little effect on 7-day survival of cetuximab (Figure 5A). For long-term selection, 5 × 10^5^ SW48 cells were treated (i) with vehicle (DMSO) control, (ii) 1 μM senexin B, (iii) 100 nM cetuximab, or (iv) 100 nM cetuximab + 1 μM senexin B. Cells were replated and the number of live cells was determined (in duplicates) by flow cytometry every 6–10 days, for a total of 81 days. Changes in cell number (calculated at each time point taking dilutions into account; see **Materials and Methods**) relative to the control cells are plotted in Figure 5B.

Senexin B alone had no significant effect on cell growth over time. 100 nM cetuximab was growth-inhibitory for the first 19 days of treatment but after that point cetuximab no longer inhibited cell growth and the cells continued to grow until the end of the study (81 days), at a rate that was even higher than the control (marked by the slope of the curve), indicating the development of a cetuximab-resistant phenotype. The addition of senexin B initially decreased response to cetuximab, suggesting an apparently antagonistic interaction until the time when cells treated with cetuximab alone developed resistance. In contrast, the combination of cetuximab with the CDK8/19 inhibitor continued to inhibit cell growth until the end of the study (day 81), with no apparent development of resistance. The difference in calculated cell numbers between cetuximab alone and cetuximab + senexin B treatments exceeded four orders of magnitude starting between days 62 and 69. Therefore, despite the apparent antagonism in the short-term, the addition of CDK8/19 inhibitor greatly improves the outcome of cetuximab treatment in the long-term, by preventing the development of cetuximab resistance.

## 4. Discussion

In the present study, we have analyzed the effects of CDK8/19 inhibition on the development of adaptive resistance to EGFR-targeting small molecules (gefitinib and erlotinib) or an antibody (cetuximab). We have carried out single-step selection for resistance to EGFR-targeting drugs by plating a relatively small cell number (1.5 × 10^5^ cells for BT474 and SKBR3 and 5 × 10^5^ cells for SW48 cells) and using relatively short periods of drug exposure (up to 10 weeks). According to recent analysis [47] mutation rates per base per cell division in cancer are on the order of 10^−8^–10^−7^ and a minimum of 2 × 10^7^ cells (10 T175 flasks) would be required to have even a single mutant; it also takes 6–12 months of selection to achieve full drug resistance. Nevertheless, in our study, resistance in all the cell/drug combinations developed in every flask in less than 10 weeks, suggesting that such resistance represented drug adaptation of cell populations and was not mediated by genetic mechanisms. In all the tested cell-drug combinations, the development of this adaptive resistance was prevented by a highly selective CDK8/19 inhibitor senexin B. The role of CDK8/19 in the development of resistance was verified through the use of another highly selective CDK8/19 inhibitor, 15w, which bears no structural similarity to senexin B. To simplify future analysis of the role of CDK8/19 Mediator kinase in drug resistance, we are currently generating a series of tumor cell lines with CRISPR/CAS9 knockout of CDK8 and CDK19, followed by re-expression of the wild-type Mediator kinases and their kinase-dead mutants.

Although CDK8/19 inhibitors consistently prevented the development of adaptive resistance to EGFR-targeting drugs in all four tested cell line/EGFR inhibitor combinations, this effect in most cases could not be attributed to drug synergy or to reversal of resistance. In particular, gefitinib-adapted BT474 cells showed the same increase in resistance to gefitinib or erlotinib as to combinations of these drugs with senexin B, although erlotinib-adapted cells were less resistant to the combination of erlotinib with senexin B than to erlotinib alone. No synergy was apparent in gefitinib or erlotinib combinations with senexin B (in contrast to a strong synergy found between erlotinib and THZ1, an inhibitor of CDK7/CDK12/CDK13 [48]). In the course of cetuximab selection, the addition of senexin B even decreased the response to cetuximab in the short-term but nevertheless prevented the development of cetuximab resistance. Hence, the observed prevention of resistance most likely reflects the inhibition of CDK8/19-mediated transcriptional reprogramming [18,34] that prevents the emergence of adaptive resistance to EGFR-targeting drugs.

Numerous mechanisms of resistance to EGFR inhibitors have been described [42,44,49,50]. These include activating mutations in EGFR itself, mutations of BRAF, KRAS and PIK3CA, as well as amplification or non-genetic upregulation of HER2 and MET, and downregulation or mutation of PTEN. The resistance was also associated with the activation of various compensatory pathways, such as Wnt/β-catenin and NFĸB, and compensatory growth factor receptor signaling, including overexpression of HER family ligands, as well as HGF and FGF. Epithelial-mesenchymal transition (EMT) has also been identified as a major mechanism of resistance to EGFR-targeting drugs [49]. As discussed above, the small cell number used in our selections and the observed rapid development of resistance argue against the involvement of any genetic mechanisms. In addition, cross-resistance of gefitinib- and erlotinib-adapted cells argues against the involvement of inhibitor-specific mutations in the EGFR gene. Since drug-resistant cell populations were isolated here via rapid single-step adaptation, they almost certainly represent mixtures of cells with different transcriptomic mechanisms of resistance. With the advent of single-cell (sc) RNA-Seq, it became clear that adaptive drug resistance involves rapid transcriptional changes, which are observed concurrently in the same cell population [5,6,7,50]. In particular, a heterogeneous pattern of resistance-associated transcriptional changes was observed in scRNA-Seq analysis of the effects of short-term (5-day) cetuximab treatment in three head and neck squamous cell carcinoma cell lines [51]. Transcriptomic heterogeneity within the same cell lines was found to increase as an immediate response to cetuximab, and this effect was suggested to indicate that different cell subclones in the same cell line are activating alternative pathways to overcome EGFR inhibition [51]. In the future, we are planning to use scRNA-Seq to identify the spectrum of CDK8/19-dependent transcriptomic mechanisms that lead to non-genetic resistance to EGFR-targeting drugs.

We have previously reported that CDK8/19 inhibitors prevent the induction of resistance-associated anti-apoptotic factors upon treatment with DNA-damaging drugs or ionizing radiation [25] and suppress the emergence of estrogen-independent ER-positive breast cancer cells upon prolonged estrogen deprivation [33]. These results, combined with the present study, indicate a pleiotropic effect of CDK8/19 on the emergence of drug resistance and suggest potential utility of CDK8/19 inhibitors in preventing the development of resistance to different classes of drugs.

## Figures and Tables

**Table 1:** IC_50_ values (µM) for parental and gefitinib- or erlotinib-adapted cell populations in 7-day growth inhibition assays shown in Figure 3 and Figure 4. In case of drug combinations, IC_50_ values are shown for each drug; fold change in IC_50_ relative to parental cells shown in parentheses (*italicized*). IC50 values for senexin B in SKBR3 cells could not be determined due to the low extent of growth inhibition.

	BT474-Par	BT474-GefR	BT474-ErlR	SKBR3-Par	SKBR3-GefR
Gefitinib	0.140	0.973 (*6.95*)	0.863 (*6.16*)	0.619	1.530 (*2.47*)
Senexin B	1.547	2.430 (*1.57*)	2.561 (*1.66*)	N/A	N/A
Senexin B + Gefitinib (1:1)	0.087/0.087	0.510/0.510 (*5.86*)	0.612/0.612 (*7.03*)	0.403	0.644 (*1.60*)
Erlotinib	2.489	7.329 (*2.94*)	8.520 (*3.42*)	2.478	3.437 (*1.39*)
Senexin B + Erlotinib (1:5)	0.351/1.753	0.473/2.124 (*1.35*)	0.915/4.577 (*2.61*)	0.258/1.292	0.328/1.641 (*1.27*)
